# Soluble isoforms of the DC-SIGN receptor can increase the dengue virus infection in immature dendritic cells

**DOI:** 10.1016/j.bjid.2024.103873

**Published:** 2024-09-25

**Authors:** Lailah Horácio Sales Pereira, Amanda do Carmo Alves, Gabriela Francine Martins Lopes, Brenda Fernandes da Silva, Mariana Sousa Vieira, Débora de Oliveira Lopes, Jaqueline Maria Siqueira Ferreira, Luciana Lara dos Santos

**Affiliations:** a Universidade Federal de São João del-Rei (UFSJ), Laboratório de Biologia Molecular, Divinópolis, MG, Brazil; b Universidade Federal de São João del-Rei (UFSJ), Laboratório de Microbiologia Médica, Divinópolis, MG, Brazil; c Universidade Federal de Minas Gerais (UFMG), Laboratório de Imunoparasitologia, Belo Horizonte, MG, Brazil

**Keywords:** sDC-SIGN, DC-SIGN, CD-209, Dengue, Viral pathogenesis

## Abstract

Dengue is a disease with a high-impact on public health worldwide. Many researches have focused on the cell receptors involved in its pathogenesis. The role of soluble isoforms of DC-SIGN (Dendritic Cell-Specific ICAM-3 Grabbing Non-integrin) receptor in the process of Dengue Virus (DENV) infection is not well understood. This work proposes to evaluate changes in the infection process of Immature Dendritic Cells (iDCs) by DENV in the presence of DC-SIGN recombinant soluble isoforms 8, 10, and 12. The recombinant isoforms were built by heterologous expression, the DENV-2 was multiplied in the *Aedes albopictus* C6/36 cells and quantified in BHK-21 cells, and the iDCs were produced from the THP-1 strain. Infection assays were performed in the presence of iDCs, DENV-2, and isoforms 8, 10, and 12 separately at 25, 50 and 100 ng/mL. The final viral load was estimated by qPCR and statistical analysis was performed by Kruskal-Wallis and ANOVA tests. The iDC profile was confirmed by increasing expression of CD11c, CD86, and CD209 surface markers and maintaining CD14 expression. Infection assays demonstrated a 23-fold increase in DENV viral load in the presence of isoforms 8 and 10 at 100 ng/mL compared to the viral control (*p* < 0.05), while isoform 12 did not alter the viral load. It was possible to conclude that at 100 ng/mL isoforms (8 and 10) can interact with DENV, increasing viral infection, and potentially acting as opsonins.

## Introduction

Among the human viral diseases transmitted by arthropods (arboviruses) dengue is considered the most important, being the most prevalent and rapidly spreading according to the World Health Organization.^1^ The etiological agent of the disease, dengue virus (DENV), belongs to the Flaviviridae family, and four serotypes, DENV-1, DENV-2, DENV-3, and DENV-4, have been identified by the International Committee on Taxonomy of Viruses.^2^

Immature Dendritic Cells (iDCs), macrophages, and circulating iDCs are considered the primary targets for DENV infection and replication after a sting by the infected vector.^3^, ^4^, ^5^ Interactions between DENV and Dendritic Cells (DCs) have been shown to be crucial for the transport of viral particles to secondary lymphoid organs and for the development of acquired immunity. These interactions also appear to act as a mechanism of deception to the immune system in which a greater number of DCs are infected in the primary organs of infection.^4^, ^5^, ^6^

Glycoprotein E, the main flavivirus surface protein, is responsible for viral binding to the DC-SIGN, a type C lectin receptor, on host iDCs and the endosome membrane through well-defined glycosylation sites.^3^^,^^7^^,^^8^ DENV/DC-SIGN binding initiates viral particle adsorption (receptor-mediated endocytosis). The glycoprotein E present in the DENV envelope interacts with the Carbohydrate Recognition Domain (CRD) on the DC-SIGN receptor, contributing to the binding and internalization of the virus in host cells.^9^ Some studies have proposed that the ‘sticky’ function of DC-SIGN is an independent function of infection, suggesting that even uninfected iDCs participate in the infection process by presenting the pathogen adhered to their cell surfaces to T-cells.^10^^,^^11^ In contrast, it has been shown by other studies that DC-SIGN adheres to and internalizes the pathogen, whereas a receptor closely related to DC-SIGN, DC-SIGNR or L-SIGN, acts only by adhering the pathogen to the surface of DC without internalization capacity.^12^

The CD209 gene, which codes for the DC-SIGN protein, has six exons, and five introns and goes through the alternative splicing process, which generates varied membrane or soluble isoforms.^12^^,^^13^ Studies have shown that the same individual can express more than one isoform of transcripts and synthesize their corresponding proteins.^14^ However the exact role of these soluble isoforms is not known. Protein isoforms range from 168 (isoform 9) to 404 (isoforms 1 and 5) amino acids.^12^^,^^15^ The complete DC-SIGN protein consists of four regions. CD209 exon I contain information for the cytoplasmic region of the protein (N-terminal). The transcripts that hold exon II contain information for the transmembrane domain, encoding mDC-SIGN isoforms. If exon II is lost, soluble isoforms (sDC-SIGN) are produced. Exons III, IV, V, and VI encode the extracellular portion of the molecule, which encompasses two domains: the neck region and the CRD.^12^

Exon III comprises seven and a half tandem repeats of nucleotide sequences, which encode the neck region of the protein.^16^^,^^17^ The tetramerization generated by the neck region also leads to the tetramerization of the CRD structure. The neck region projects CRD beyond the cell surface and gives DC-SIGN flexibility comparable to that of immunoglobulins, allowing it to bind to antigens on viral surfaces at different distances.^18^ The variation in expression levels and isoforms generated between individuals may have important implications for dengue immunopathogenesis.

The function of soluble isoforms is not well known; some studies have demonstrated the importance of these recombinant DC-SIGN isoforms in blocking Human Immunodeficiency Virus (HIV),^19^ DENV^20^ and Cytomegalovirus (CMV)^21^ infections. Soluble DC-SIGN isoforms are known not to have the same functional activity in terms of binding to ICAM-3 in CD4 T lymphocytes. A study with one sDC-SIGN isoform showed that it was not secreted and was located in the cytoplasm of producer cells with unknown function.^22^

In order to better clarify the function of soluble isoforms in the process of DENV infection in iDCs, this study deals with three recombinant soluble DC-SIGN isoforms. The complete recombinant soluble isoform (sDC-SIGN1B type I – isoform 10), an isoform without CRD alteration but with neck region changes (sDC-SIGN1A type III – isoform 8), and an isoform with changes in CRD, neck region, and other regions (sDC-SIGN1B type III – isoform 12) were built. Their ability to bind in mannose residues was verified previously.^23^ The choice of these three isoforms aimed to represent the variation of the expression existing in the human organism and its possible functions in the infectious process.

## Material and methods

### Protein expression, purification, purity, and function analysis

Recombinant sDC-SIGN isoforms 8, 10, and 12 were built by heterologous expression. The nucleotide sequences were obtained from GenBank, synthesized, and cloned into expression vectors with sequences encoding a histidine tail. *Escherichia coli* BL21 Rosetta DE3 cells (Novagen) were used for protein expression. The *E. coli* cells correctly transformed by protein expression vector were grown in 2XYT, at OD_600_ = 0.6. The expression was induced with isopropyl-β-d-thiogalactoside for 4 h, when bacteria were collected and lysed by ultrasonic treatment. After, recombinant proteins expressed were treated with urea 6 M to eliminate inclusion bodies, and then denatured recombinant proteins were refolded in a refolding buffer (pH = 7.4). Then, proteins were purified by affinity chromatography in a HiTrap column (GE Healthcare) and its function was confirmed by affinity chromatography in a mannose-agarose column. Purified recombinant soluble proteins were resolved by SDS-PAGE followed by western blotting.^23^

### Cells, viruses, antibodies, and cytokines

Human peripheral blood acute monocytic leukemia cells THP-1 (ATCC; number TIB-202) were cultured in RPMI 1640 medium (Gibco, Brazil) supplemented with 10 % FBS and 0.3 % Penicillin-Streptomycin-Amphotericin (PSA) B solution (Sigma-Aldrich, USA) maintained in a humidified atmosphere oven with 5 % CO_2_ at 37 °C. The continuous line C6/36 cells, obtained from *Aedes albopictus* were grown in Leibovitz L-15 medium (Cultilab, Brazil) supplemented with 10 % FBS (Sigma-Aldrich, USA) and PSA. The culture was incubated in a Biochemical Oxygen Demand (BOD) oven at 28 °C until reaching about 90 % confluency in the flask.

The C6/36 cells monolayer was infected with a Multiplicity of Infection (MOI) of 0.01 and the culture was incubated in a BOD oven at 28 °C for around four to seven days until the appearance of a Cytopathic Effect (CPE).

The titer of DENV was determined in Baby Hamster Kidney (BHK-21) cells (ATCC CCL-10) obtained from continuous lineage from the Department of Microbiology of the Federal University of Minas Gerais. The virus titer was measured by tissue culture infectious doses (TCID_50_/mL), calculated using the Reed-Muench method.^24^

The antibody was obtained from BD Biosciences, it used anti-human CD14 (PE-Cy7), anti-CD86 (PERCP-Cy5.5), anti-human CD209 (Pacific Blue), anti-CD11c (FITC), rh GM-CSF, and rhIL-4 (BD ‒ Biosciences, USA).

### iDCs differentiation from THP-1 cells

For dendritic cell differentiation, 10^4^ cells of THP-1 were plated per well in a 96-well plate, with RPMI medium supplemented with 10 % BFS and cytokines for differentiation, GM-CSF (50 ηg/mL; Immunotools), and IL4 (50 ng/mL; Immunotools). The cells were incubated in a BOD with 5 % CO_2_, at 37 °C for seven days. At each 72 h the differentiation, the medium, and the cytokines were renewed.

### Phenotyping of generated iDCs

For phenotypic determination of iDCs by flow cytometry, the cells were stained with anti-CD11c, anti-CD86, anti-CD209, and anti-CD14. The acquisition was performed in the flow cytometer using a LSRFortessa with the software FACSDiva (BD Biosciences), in the Interdisciplinary Laboratory of Human Diseases Research, in the Department of Clinical and Toxicological Analysis of the Faculty of Pharmacy of UFMG. Data were analysed with FlowJo 10.0 software (Tree Stars Inc.).

### Infection assays

The iDCs obtained were used in the DENV-2 infection assays in the presence of recombinant sDC-SIGN isoforms 8, 10, and 12 at concentration of 25, 50 and 100 ng/mL. The assays evaluated the overall activity of the isoforms on the viral particles.

The cells were infected on the last day of differentiation. Around 10^6^ cells/well were infected with DENV-2 at 5 × 10^7^ virus/mL and a MOI of 1. The infection assay was made using the following steps: A) Plate 1 (Protein + iDCs): The iDCs were incubated with 25, 50 or 100 ng/mL of each protein, separately, for 30 min at 37 °C and 5 % CO_2_, in a total volume of 150 μL of 5 % RPMI. B) Plate 2 (Protein + DENV): The recombinant proteins (25, 50, or 100 ng/mL) were incubated with DENV-2 for 30 min in the same conditions as Plate 1. C) 150 μL of Plate 2 was transferred to the cell plate (Plate 1) which was incubated at 37 °C and 5 % CO_2_ for two days. According to Alen et al.,^25^ the peak of infection occurs in 48 h. D) After 48 h of infection, the supernatant from each well was collected and the extraction of viral RNA, Reverse Transcription (RT-PCR), qPCR of cDNA, and ultrafreezer storage were made. Experiments were performed in duplicate. All processes were repeated three times in different months to evaluate the reproducibility of the results.

### qPCR

The viral load was measured by absolute qPCR of DENV-2. The DENV-2 RNA obtained from the cell culture supernatant was extracted with a High Pure Viral Nucleic Acid Kit (Roche, Switzerland) according to the manufacturer's guidelines. The viral RNA was quantified in an absorbance spectrophotometer and submitted to the RT-PCR in a thermocycler (AB-Applied Biosystem, Veriti thermal cycler, 2010). The cDNA production was performed from approximately 1 ug of RNA, 10× Random Primer, 10 mM dNTP's, Depc water, 10× RT enzyme buffer and the MultiScribe® Reverse Transcriptase enzyme with a High Capacity cDNA Reverse Transcription Kit (Applied Biosystems, USA). The reaction conditions for cDNA synthesis were: 01 cycle at 94°C for 1-minute; 30 cycles comprising three steps of 94°C for 30 seconds, 57°C for 30 seconds, and 72°C for 30 seconds, and 01 cycle at 72°C for 10 minutes.

The cDNA was serially diluted (10^−1^ to 10^−10^) to be used in a subsequent qPCR to build a standard curve with primers as previously described: Forward primer (F) 5′-TTA GAG GAG ACC CCT CCC-3′ and reverse primer (R) 5′-TCT CCT CTA ACC TCT AGT CC −3′.^26^ In the qPCR 2 μL of diluted cDNA, 10 pmoL of each primer, and 5 μL of the HOT FIREPol® solution EvaGreen® qPCR Supermix (Solis Biodyne, Estonia) were used. The amplification conditions were: 01 cycle at 95°C for 12 minutes, 40 cycles comprising three steps of 95°C for 15 seconds, 56°C for 20 seconds, and 72°C for 20 seconds. The analyses of the Tm curves included in the qPCR were performed by a denaturation step at 95°C for 15 s followed by 60°C for 1 m and a ramp up to 94°C at a rate of 0.1°C/10 s with continuous fluorescence measurement.

### Statistical analysis

Data analysis was performed using the GraphPad Prism version 7.04 statistical program. Non-parametric Kruskal-Wallis tests were used for pre-testing and Dunn's for post-testing. Normally distributed samples were evaluated by ANOVA in a pre-test and Tukey's in a post-test. The significance interval of *p* < 0.05 was considered for both tests.

## Results

**Fig. 1:**
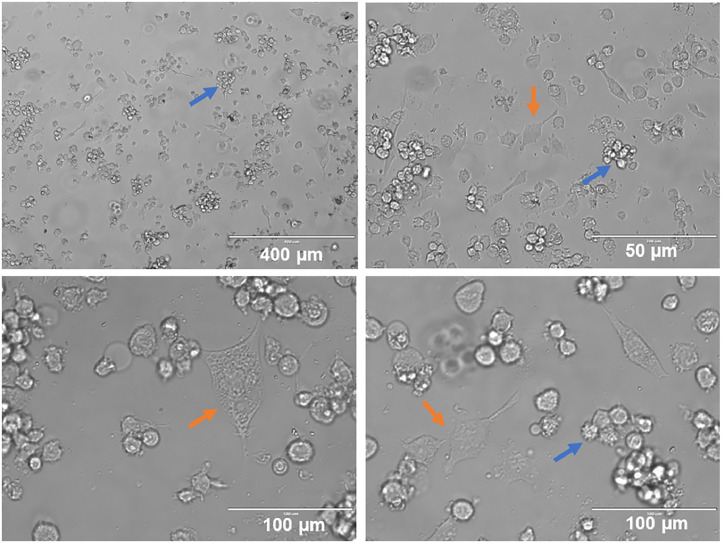
iDCs cells differentiated from THP-1 cells after 144 h. (A to D) Suspended cell clusters are observed with spicules (blue arrows) and other adhering cells with elongated conformation (orange arrows), both typical of iDCs.

In the present study, a portion of THP-1 cells differentiated for six days (144 h) with 50 ng/mL of IL-4 and 50 ng/mL of GM-CSF showed morphological changes under inverted light microscopy when adhered. Another portion that remained in suspension presented minor morphological changes. Cell clusters possibly containing undifferentiated THP-1 cells and non-adherent iDCs were observed. Differentiated iDCs, which adhered and exhibited an elongated morphology with dendrites, were also seen (Fig. 1).

**Fig. 2:**
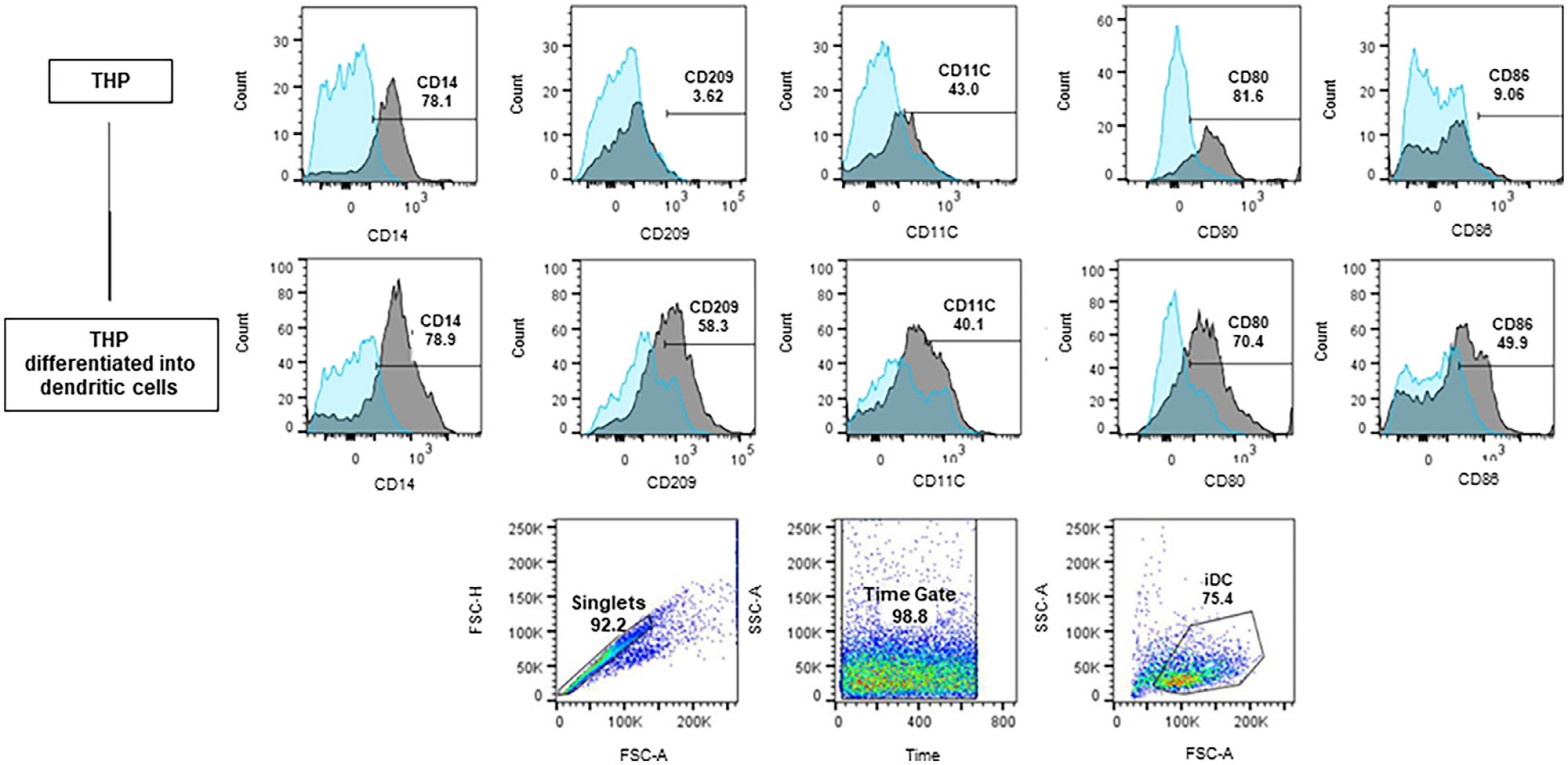
Differentiation of THP-1 into iDC. Histograms and Dotplots obtained by flow cytometry. Blue histograms ‒ unmarked isotypes; Grey histograms ‒ marked isotypes. The bars show the displacement of the marked population in relation to the unmarked one. Before differentiation, among the labeled cells (iDCs) 58.3 % expressed the CD209 receptor, 50 % expressed the CD86 receptor, and 40.1 % expressed the CD11c receptor. The joint expression of these three receptors demonstrated a profile closer to that of iDCs.

The representative analysis of the phenotyping of iDC generated from the THP-1 cells and the percentages of each surface marker in iDCs can be viewed in Fig. 2. Only singlet cells were considered for analysis and the gates strategy is also demonstrated. The gates were defined from unmarked controls and considered 10,000 events. Cells read too early or too late were disregarded by the Time Gate to avoid reading possible equipment bubbles or debris. iDCs cells (75.4 %) showed up regulation of CD86, CD209, and CD11c markers when compared to the THP-1 cells.

After obtaining DENV-2, producing the three isoforms by heterologous expression, and generating the iDCs cells, the DENV-2 iDC infection assays were performed in the presence of soluble isoforms. The experiments were performed at the concentrations of 25, 50, and 100 ng/mL of each isoform and a MOI of 1, chosen according to the reference literature. It was possible to quantify the replicates of infection and the final values obtained by qPCR reactions. These data are represented by the average of the results obtained in the three different infection assays. To cover the viral concentration range found in the samples from the infection assays, the most diluted points of the standard curve were considered. Higher mean viral concentration rates were observed just in samples containing isoforms 8 and 10 at 100 ng/mL when compared to the mean viral concentration rate of the viral control. The values obtained can be observed in Table 1.

**Table 1:** Mean concentration and viral load obtained from infection assays with 100 ng/mL of sDC-SIGN soluble isoforms.

Isoform (100 ng/mL)	Viral average concentration (ng/µL) by isoform concentration	Nº viral copies/µL by isoform concentration
8	8.3E-01^a^	7.8E+21^a^
10	5.4E-01^a^	5.1E+21^a^
12	6.0E-02	5.9E+20
Viral Control	3.0E-02	2.7E+20
Cellular Control	0.0E+00	0.0E+00

a Statistical significance compared to viral control. - There was a significant difference between all cellular and viral controls. - There was a significant difference between all samples and the cell control. - The means obtained by qPCR from replicates of infection assays are shown. - The number of viral copies (copies/mL) found in the samples was calculated using the formula: Copies/µ*L* = [g/µ*L* of RNA × 6.022‒1023]/[*transcript bp* × 660 g/moL] * Average MM of 1 bp of DNA = 660 g/moL; 1 moL = 6.02×10^23^ molecules.

**Fig. 3:**
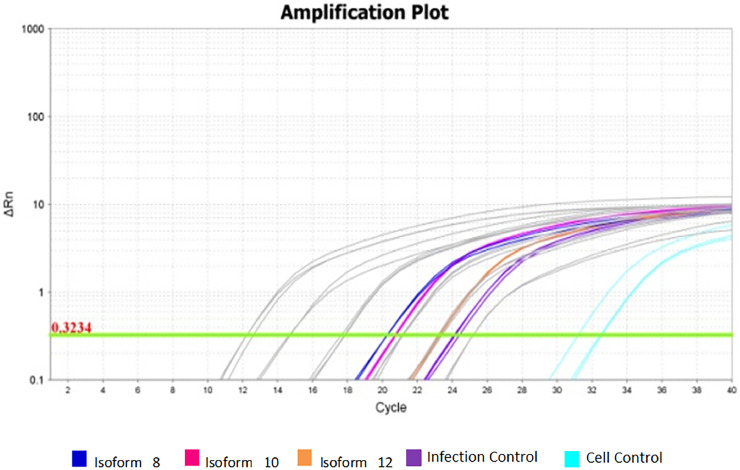
Viral amplification in samples treated with the three soluble isoforms at 100 ng/mL. Amplification plot of assays for isoforms 8, 10, and 12 at 100 ng/mL concentration. Grey: Standard curve dilutions from 10^−5^ to 10^−9^. Navy Blue: Sample treated with isoform 8 at 100 ng/mL. Rose: Sample treated with isoform 10 at 100 ng/mL. Orange: Sample treated with isoform 12 at 100 ng/mL. Purple: infection controls from assays. Light blue: cellular controls of infection assays.

**Fig. 4:**
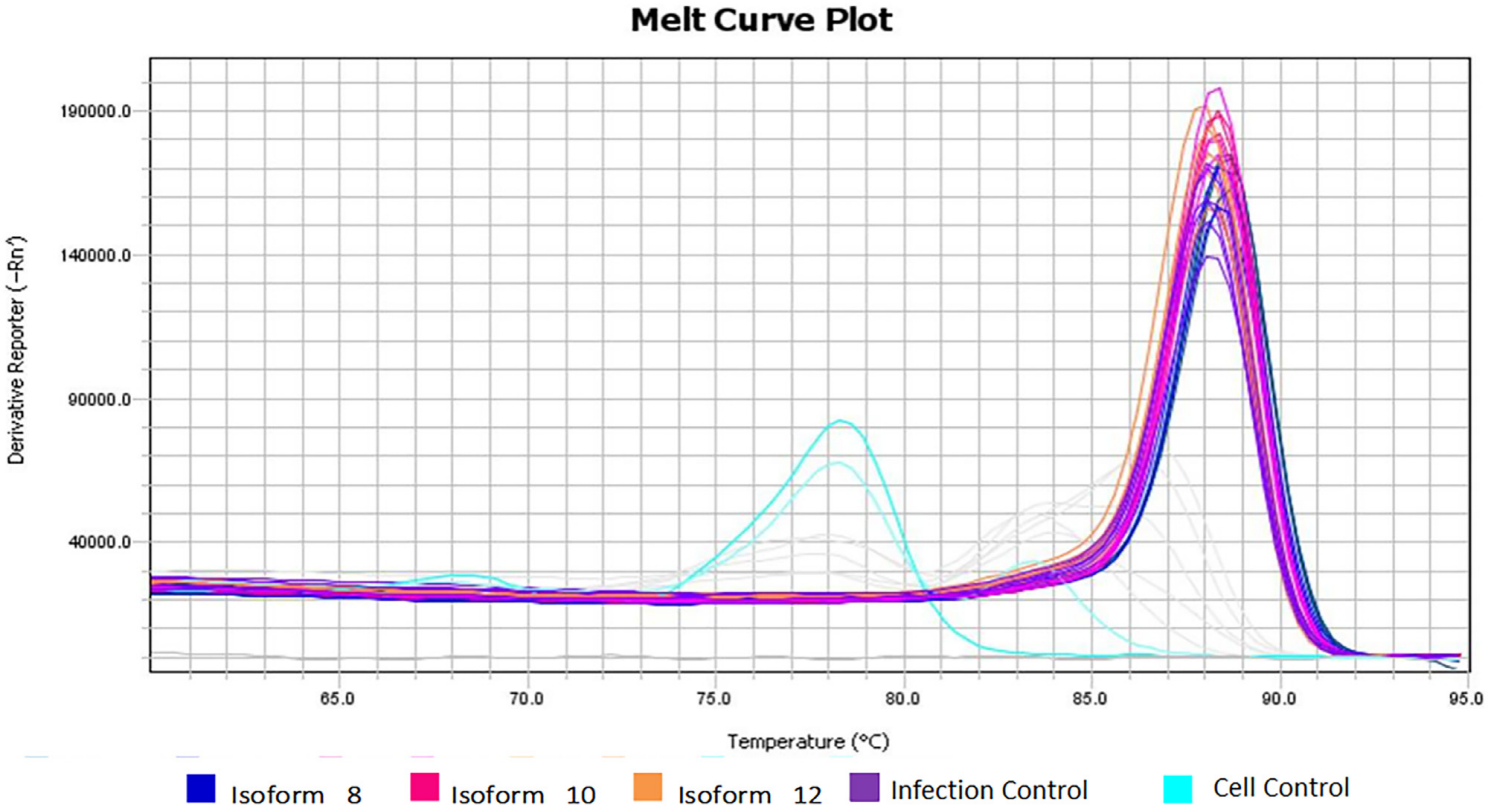
Different Tm dissociation curve between cell control and other virus-containing samples. The melting temperature found for the cell control samples (light blue) differs from the amplification temperature of the DENV-2 fragments, confirming that the fluorescence detected in the cell control was equivalent to primer dimers and not to amplification of viral fragments.

There was a statistically significant difference between cellular and viral controls (*p* < 0.05) (Table 1). The cellular control presented a high Ct (Fig. 3), but a different Tm dissociation curve from the virus-containing samples, and there was no amplification in the negative control (Fig. 4).

The sample containing the isoform 12, with altered CRD, at 100 ng/mL presented a similar mean viral concentration to that found in the viral control. The same happens in the concentration of 25 and 50 ng/mL. Fig. 3 shows the amplification graph of samples obtained from the infection assay at 100 ng/mL.

## Discussion

The differentiation results are similar to those described by Guo et al.^27^ with the phenotyping characteristics and morphology equating to Berges et al.^28^

As a monocytic lineage, THP-1 cells must have high CD14 expression; as observed in the literature, when differentiated with IL-4, they continue to show high CD14 expression.^29^ In contrast, THP-1 cells have low CD86 and CD11 expression, as expected in monocytic strains,^30^ and iDCs show higher expression of these markers.^31^ In addition, THP-1 has a variable (uptrend) expression of CD209, which should increase in iDCs.^29^^,^^32^

As predicted, not all iDC differentiated from the leukemia cell model showed elongated phenotype although most cells presented changes in round to slightly longer elongated conformation, as described in the literature.^28^ This fact can be explained by the reasoning that 100% cell differentiation does not occur and possibly, because there are non-adherent suspended iDCs, this gives them a rounded shape when viewed under inverted light microscopy.

THP-1 cells have been standardized as an easy, fast, and reliable model for iDC differentiation, as corroborated by the literature.^29^^,^^30^ Chan et al.^33^ differentiated THP-1 on iDC for five days with an initial density of 1 × 10^6^ cells per well with 40 ng/mL IL-4 and 40 ng/mL GM-CSF at 37 °C in a humidified environment with 5 % CO_2_. Cytokines were changed on the third day and cells were labeled at the end of the fifth day. At the end of differentiation, THP-1 cells showing intracellular IL-10 expression, de novo expression of the CD86 costimulatory molecule, and surface receptors indicative of iDCs CD11c, CD40, and CD209 were observed. In addition, the cells did not show the expression of the marker CD83, an indicator of iDC maturation. Under these conditions, a THP-1 iDC differentiation rate of 75.4 % was obtained and the cells acquired functional properties of iDCs, such as increased receptor macromolecular endocytosis and low T lymphocyte stimulatory capacity.

As already reported in the literature, IL-4 and GM-CSF are sufficient to promote differentiation into functional iDC.^28^^,^^33^ After differentiation, cells maintained the phenotypic properties of iDC for approximately six weeks by changing cytokine differentiation factors every three days.^28^ These data based this work on the choice of differentiation and concentration factors, and differentiation time and surface markers observed at the end of differentiation.

Regarding mean concentration and viral load obtained from infection assays with 100 ng/mL of sDC-SIGN soluble isoforms, the supposed amplifications that occurred in the cell control samples did not correspond to the viral amplification, since these samples contained only cells and PBS. The experiments were performed with the Eva green fluorophore, which interleaves on any double strand, not being specific. In this case, this incorporation clearly occurred in the primer dimers used in the reaction that was not consumed, since the virus was not present. This hypothesis can be confirmed by the melting curve shown in Fig. 3, showing a different Tm for these samples (light blue curves) and non-amplification of the viral fragment.

Higher mean viral concentration rates were observed just in samples containing isoforms 8 and 10 at 100 ng/mL when compared to the mean viral concentration rate of the viral control. Mean viral concentrations in these samples (Table 1) were approximately 23 times higher than in the viral control. There were noteworthy increases at 28 times higher for isoform 8 and 18 times higher for isoform 10, and the difference in infection rate between isoforms was also significant (*p* < 0.05).

One initial hypothesis to explain the increase in the viral load when soluble isoform 8 and 10 are used is the possible conjugation of recombinant soluble isoforms with membrane isoforms, already existing in iDCs, that have not yet formed stable (di-, tri-, or tetra-) multimers.^21^^,^^34^ This conjugation could contribute to increasing the infection, since the tetrameric structures of DC-SIGN, which increase the stability of the DENV binding and other ligands, would be increased. Given the results, we observed that the above theory may have occurred: isoforms 8 and 10 associated with membrane isoforms of differentiated cells forming multimers would increase the avidity by circulating DENV with a consequent increase in the rate of infection. This result was similar to that found by Plazolles et al.,^21^ who demonstrated increased CMV infection in the presence of recombinant sDC-SIGN. When they performed CMV infection testing on monocyte-derived Dendritic Cells (moDC) with MOI = 1, for 24 to 48 h in the presence of decreasing amounts of soluble isoforms 6 (sDC-SIGN1A type I) and 8 (sDC-SIGN1A type III) (400 to 12.5 ng/mL), they observed about double the infected DCs than in infection control, with a concentration between 100 ng/mL and 50 ng/mL of protein. Concentrations greater than 100 ng/mL and less than 50 ng/mL of the sDC-SIGN1A type I recombinant isoform 6 showed no difference in infection rate.

Other studies have shown blockade of *S. aureus* and HIV infection in the presence of soluble DC-SIGN isoforms. Kwon et al.^19^ and Navarro-Sanchez et al.^35^ suggested DC-SIGN protein increases viral HIV and DENV infection only when expressed in the cell membrane. In both studies, there was blockade of infection by sDC-SIGN with MOI variable from 5 to 10; however, only CRD was produced and considered as soluble DC-SIGN. Kwon et al. further demonstrated that mDC-SIGN with the truncated cytoplasmic domain region is capable of capturing circulating viruses but is unable to internalize them with low MOI.^19^ This corroborates the highlighted importance in our study that isoforms be fully studied, as they exist *in vivo*, because all portions of the protein perform functions that are still being discovered.

Another possible justification for the results found in the present work is that at high concentration (100 ng/mL) there is bioavailability of sDC-SIGN that complexes rapidly but inefficiently to circulating viral particles acting as opsonins rather than infection blockers. Thus, soluble isoforms at high concentrations could lead to a high number of immobilized viral particles to capture and internalize by iDCs, favoringfavouring infection. As already described by Mikloska et al.,^36^ this hypothesis presents the need for another receptor that favors iDC opsonization, such as CD11b has been shown to facilitate HIV opsonization by iDC mDC-SIGN-dependence. It is also possible that these aggregates (virus + sDC-SIGN) may associate with mDC-SIGN and somehow facilitate viral penetration into cells. These data indicate that the virus ‘opsonized’ by sDC-SIGN is more effectively captured by iDCs than free viruses. sDC-SIGN molecules capable of interacting with infectious agents at high serum concentrations could potentiate the severity of diseases such as Dengue, since infection itself can alter the expression pattern of isoforms.^21^^,^^36^

DC-SIGN molecules in tetramers are known to bind to better affinity N-glycan residues, with the neck region of the protein essential in this oligomerization.^3^^,^^14^ Although isoform 8 has unchanged CRD, its neck region is altered and it is well demonstrated that it is through this region that interaction with other soluble and membrane isoforms occurs.^14^ Even so, in our results a significant increase in infection was also found in the trials with the presence of this isoform (Table 1). One possible explanation for this is that remaining amino acid residues in the neck region of isoform 8 (three and a half tandem repeats) are sufficient to promote interaction with other neck regions of membrane isoforms also forming the multimers responsible for the increase of avidity for DENV-2 and, consequently, increasing the infection rate. Moreover, because it is a smaller molecule than the complete isoform, isoform 8 linked to DENV could be more easily internalized.

The second hypothesis was that recombinant soluble isoforms capable of binding to DENV could interact with each other to form stable multimers that could neutralize circulating viral particles and, consequently, the binding, internalization, and infection of iDCs. This hypothesis was discarded in experiments with isoforms 8 and 10 at 100 ng/mL.

Besides the possibility of observing this process *in vitro* assays, these events could also occur with circulating sDC-SIGN, naturally or via therapeutic administration, neutralizing the infection, and therefore, it is important to observe the necessary concentrations of circulating isoforms to provide blockade. Schmid and Harris^37^ proposed that the skin is an important site for therapeutic actions or even for intradermal vaccination, since DCs and macrophages are the primary target of DENV infection with additional monocyte recruitment for further differentiation into iDCs susceptible to infection and subsequent antigen presentation.

DENV causes a diverse spectrum of disease ranging from asymptomatic infection and mild febrile illness to more serious complications, including hemorrhage and shock. The associations between host genetics, DENV infection and clinical outcome are complex and may involve more than one factor such as age, ethnicity, primary or secondary infection, patient's metabolic conditions and even genetic factors that lead to the expression of proteins involved in the process. of infection.^38^^,^^39^

Studies demonstrate the relationship between the severity of the disease and the different polymorphism profiles of genes that express proteins associated with DENV infection, such as DC-SIGN.^40^^,^^41^ This demonstrates the importance of the protein structure of receptors for viral infection. Therefore, therapeutic strategies targeting protein structures involved in the DENV infection process, associated with a higher degree of infection, as observed in the present study for DC-SIGN isoforms 8 and 10, are promising.

Infection experiments in the presence of soluble isoform 12 presented different results from other isoforms. This isoform does not appear to interact with circulating viral particles at any concentration, neither increasing nor decreasing the infection rate compared to the infection control. The results obtained were statistically similar to those found in viral control. This result is in agreement with those found in the mannose column binding experiments which demonstrated the inability of this soluble isoform to bind to these residues.^23^ Thus, isoform 12 would be unable to bind to DENV glycoprotein E, apparently not interfering in any way with the infection process.

Unlike isoforms 8 and 10, isoform 12 has an altered CRD region, which is essential for the binding and internalization of DENV in the cell.^9^ Therefore, it can be inferred that the absence of this region prevents the interaction of the virus with isoform 12, maintaining infection levels similar to those of the viral control.

Finally, an important aspect to be analysed in new studies is the relationship between viral infection and variation at the expression level of soluble isoforms, since we observed that infection rates were increased at the tested concentration, which may suggest a mechanism to ‘aid’ viral particles.

## Conclusion

DC-SIGN soluble isoforms with intact CRD (8 and 10) studied in this work maintain the ability to bind to DENV mannose residues and potentiate infection rates in 100 ng/mL iDCs.

DC-SIGN soluble isoform 12 with altered CRD lost its ability to interact with DENV mannose residues and did not generate significant changes in mean viral load at the concentration tested.

The amino acid residues that constitute the neck region seem to allow the polymerization of the isoforms, even in smaller repetitions than in the canonical isoform. Isoform 8, which has an altered neck region but intact CRD, was also able to increase the rate of infection. In addition, the smaller size of this isoform seems to favor the internalization of the sDC-SIGN-DENV complex.

## Funding

This study was funded by the Coordenação de Aperfeiçoamento de Pessoal de Nível Superior ‒ Brazil (CAPES) ‒ Funding Code 001, Conselho Nacional de Desenvolvimento Científico e Tecnológico (CNPq) and Fundação de Amparo à Pesquisa do estado de Minas Gerais (FAPEMIG).

## CRediT authorship contribution statement

**Lailah Horácio Sales Pereira:** Conceptualization, Investigation, Methodology, Software, Validation, Visualization, Writing – original draft, Writing – review & editing. **Amanda do Carmo Alves:** Investigation, Methodology, Writing – review & editing. **Gabriela Francine Martins Lopes:** Methodology, Validation, Writing – review & editing. **Brenda Fernandes da Silva:** Methodology, Validation, Writing – review & editing. **Mariana Sousa Vieira:** Methodology, Validation, Writing – review & editing. **Débora de Oliveira Lopes:** Funding acquisition, Supervision. **Jaqueline Maria Siqueira Ferreira:** Conceptualization, Formal analysis, Funding acquisition, Investigation, Methodology, Project administration, Resources, Supervision, Validation, Writing – original draft, Writing – review & editing. **Luciana Lara dos Santos:** Conceptualization, Data curation, Formal analysis, Funding acquisition, Investigation, Methodology, Project administration, Resources, Software, Supervision, Writing – review & editing.

## Conflicts of interest

The authors declare no conflicts of interest.
